# Understanding How Relational Health Effects Intimate Partner Violence Perpetration among Low-Income, Black, Indigenous, Men of Color Exposed to Adverse Childhood Experiences: An Exploratory Study

**DOI:** 10.3390/ijerph18083890

**Published:** 2021-04-08

**Authors:** Laura A. Voith, Hyunjune Lee, Katie N. Russell, Amy E. Korsch-Williams

**Affiliations:** Center on Trauma and Adversity, Jack, Joseph, and Morton Mandel School of Applied Social Sciences, Case Western Reserve University, Cleveland, OH 44106, USA; hxl809@case.edu (H.L.); knr25@case.edu (K.N.R.); axk130@case.edu (A.E.K.-W.)

**Keywords:** intimate partner violence, mixed methods, adverse childhood experiences, socioeconomic disadvantage, social support

## Abstract

Relational health has emerged as a consistent factor that can mitigate the effects of trauma among children; however, less is known about relational health with adults, particularly related to intimate partner violence (IPV) perpetration among racially and socioeconomically marginalized men. The Exploratory Sequential Design, Taxonomy Development Model was used. Semi-structured interviews (*N* = 11) and narrative analysis were conducted in Phase I. In Phase II, variables approximating the key themes that emerged in Phase I were selected from an existing dataset (*N* = 67), and relationships were examined using bivariate associations. The sample consisted of low-income Black, Indigenous, men of color (BIMOC) in a batterer intervention program (BIP). Adverse life experiences shaped participants’ world view via mistrust in others, stifling emotions and vulnerability, and a sense of personal guilt and shame. These orientations were then carried into adult relationships where men coped using social isolation to manage challenges, negatively affecting intimate relationships. For some men, mental health exacerbated these circumstances. Significant bivariate and multivariate associations supported this narrative. This study lays the foundation for future research to examine the potential effects of social support on IPV perpetration. BIPs should consider augmenting programming to enhance men’s social networks to support their use of nonviolence after program completion.

## 1. Introduction

Stemming from systemic racism and poverty, Black, Indigenous, and youth of color face an unequal burden of adversity and trauma in the United States [1,2]. Traumatic events (e.g., violent victimization or witnessing violence) during childhood have been linked to an array of negative outcomes (e.g., physical, psychological, cognitive, and behavioral) in later developmental stages, including adulthood (e.g., [3,4]). Within the scope of this research, numerous studies have linked childhood trauma with intimate partner violence (IPV) perpetration and victimization in adulthood (see [5]). IPV includes physical violence, sexual violence, stalking, and psychological abuse between two current or former intimate partners [6]. Prevalence rates of IPV estimate that 1 in 3 women experience some form of IPV victimization in their lifetime, with multiracial, Black, and Indigenous women experiencing disproportionately higher rates compared to Latina, White, and Asian women [6]. Despite the robust body of research examining the link between childhood trauma and adulthood outcomes such as IPV perpetration, less research examines factors that might mitigate that relationship especially among low-income, Black, Indigenous, Men of Color (BIMOC) involved in the criminal justice system. Relational health—the presence of attuned and caring caregivers, family members, mentors, teachers, and community members—has consistently emerged as a factor that can diminish the effects of trauma, particularly among children [7,8]. However, less is known about how relational health operates with adults, particularly as it relates to IPV. Most research on the relationship between childhood trauma, relational health, and IPV focuses on how social support and isolation affects victims of IPV in adulthood [9], with little research examining how it might affect IPV perpetration in adulthood. Understanding if and how relational health affects the relationship between childhood trauma and IPV perpetration among socioeconomically disadvantaged adult men in batterer intervention programs (as a requirement of probation) may shed light on new pathways to intervene and ultimately contribute to the cessation of IPV perpetration in disproportionately affected communities.

## 2. Theoretical Orientation

Drawing from trauma theory, trauma can be defined as an event, or series of events, that is experienced by an individual as physically or emotionally harmful or life threatening, and can have lasting effects on functioning and well-being [10,11]. The way in which an individual experiences a traumatic event can be connected to a range of factors, such as cultural and religious beliefs, family and social supports, and developmental stage [10,12,13,14]. Traumatic experiences can cause a substantial shift in world view, disrupting one’s sense of safety, perception of self, and trust in others [14,15]. Negative appraisals of self and others persisting over time contribute to a sense of ongoing threat, which is associated with the activation of symptoms including avoidance, hyperarousal and re-experiencing of traumatic memories [16,17].

Experiences in early life determine the organization and functioning of the adult brain [8]. Exposure to traumatic interpersonal adversity can significantly alter cognitive, social, psychological and biological development in children, resulting in increased dysregulation of impulses and affect, increased inattention, disturbances of attribution and schema, and relational difficulties [14,18,19]. Early life adversity has been connected with enduring alterations in brain–body signaling, leading to physical symptoms, emotional dysregulation, and altered threat appraisal and stress responses in adulthood [20,21].

Research demonstrates that the consistent presence of a caring adult or “relational health” is a primary protective factor that can mitigate the effects of adversity and trauma [8]. Social support serves as a buffer from the pathogenic influence of trauma by strengthening perceived coping abilities, reducing negative reappraisals of traumatic experiences, and decreasing physiological responses to trauma [22,23,24,25]. The presence of positive caregiver and adult role model figures in childhood has been shown to predict individual resilience late in life [26].

Positive relational interactions mitigate activation of stress responses and produce regulating effects that are central to healing, recovery and healthy development after traumatic experiences [7]. The presence of consistent, attuned interactions with caregivers and other caring adult figures becomes a mediator of individual stress response baseline and reactivity, producing modulating effects. Conversely, inconsistent relationships (i.e., relational poverty) or harmful relationships can activate responses that ultimately exacerbate trauma. Extending this to adulthood, we infer that social support from family, friends and community may have ameliorating effects for adults recovering from childhood trauma and adversity and that relational poverty or harmful relationships may preserve the long-term effects of childhood trauma and adversity.

### 2.1. Literature Review

#### 2.1.1. Effect of Childhood Trauma on Adult Mental Health and IPV

There is a substantial body of literature that examines the effect of childhood trauma on adult mental health, with most studies focusing on outcomes such as chronic depression, posttraumatic stress disorder (PTSD), substance abuse, and other mental health disorders [27,28]. Another robust body of research examines the relation between mental health and IPV perpetration (e.g., [29]). For instance, one study found that substance abuse and depression independently predicted IPV perpetration, with depression also predicting IPV victimization among a sample of primarily Black and Hispanic men and women in an urban city [30]. Researchers have begun linking these two separate bodies of research, examining the link between childhood trauma, the mediating effects of mental health, and IPV. For example, Machisa and colleagues found a mediating effect of PTSD symptoms on the relation between childhood trauma and IPV perpetration among a sample of South African men [31]. A recent study of men enrolled in IPV intervention programs reported an indirect association between traumatic childhood experiences and IPV perpetration via PTSD symptomology and symptoms of dominance [32].

#### 2.1.2. Effect of Social Support and Isolation on Health and Well-Being

The positive effects of social support on individuals’ physical and mental health and well-being has been well-documented in the literature [33]. Greater social support has been linked with decreased depression and anxiety symptoms [34], physical health-related quality of life [35], and resilience in violence-exposed individuals [36,37]. A systematic review of 40 systematic reviews (i.e., an “overview”) focusing on social isolation and loneliness (Leigh-Hunt et al., 2017) found consistent evidence of the link between social isolation and loneliness and some mental health outcomes. For example, researchers reported that larger and more diverse social networks with high quality relationships protected against depressive symptoms [38]. Another study found that socially isolated individuals who report stressful events as more “intensely stressful” are more likely to avoid or passively cope with those events, and exhibit less effective physiological healing and maintenance from stressful events [39].

#### 2.1.3. Effect of Social Support and Isolation on IPV

Of the research examining the effect of social support and isolation on IPV, the majority focuses on victimization. Social support has been linked to improved psychological well-being following IPV victimization [9], as well as reduced revictimization [40]. Furthermore, studies show that social isolation (i.e., lack of social support) is associated with continued IPV victimization, though this has been primarily examined among samples of women (e.g., [41]). Less research has examined the effects of social support and isolation on IPV perpetration. Of the research that does exist, most studies focus on the link between IPV perpetration and social networks that support violence (i.e., violent peers, support network gender stereotypes; [42,43]). To our knowledge, no studies have examined the potential protective effects of social support and damaging effects of social isolation on IPV perpetration.

### 2.2. Gaps and Current Study

There is a robust body of research linking adverse childhood experiences (ACEs) and trauma with numerous deleterious outcomes in later stages of development; however, comparatively less research exists on protective factors that can ameliorate the long-term effects of early adversity on outcomes later in life, especially focusing on IPV. Theory and empirical research support the notion that relational health can promote resilience and healthy development for children in the face of adversity [8]. Extending this theory to adulthood, social support as a form of relational health has shown protective effects for those vulnerable to IPV victimization. However, less is known about how social support protects against IPV perpetration or how social isolation may enhance the risk of IPV perpetration. Addressing this gap could help to inform more effective intervention practices in the field of IPV.

The current study explored the association between childhood trauma, social support, and IPV perpetration using mixed methods with a sample of low-income Black, Indigenous, Men of Color (BIMOC) in a batterer intervention program (BIP) as a requirement of probation. The study was conducted in two phases and was guided by the following research questions: (1) How has childhood trauma shaped relationships with others among men with recent histories of IPV; (2) How does social support affect men’s relationships with their intimate partners; and (3) Are the key factors identified in men’s narratives (i.e., certain types of childhood trauma, mistrust of others, mental health, guilt/shame, and suppressed emotions) associated with social support and IPV perpetration?

## 3. Methods

### 3.1. Design

Due to limited guiding frameworks or theories to understand the potential relation between social support and IPV perpetration among men with histories of trauma, an exploratory mixed methods design was used. The Exploratory Sequential Design [44], Taxonomy Development Model [45,46] prioritizes the application of qualitative data collection and analysis (Phase I) to identify quantitative variables and hypotheses to test (Phase II). Specifically, semi-structured interviews and narrative analysis were conducted in Phase I resulting in several key themes (research questions 1 and 2), which then informed the factors examined for the quantitative analysis carried out in Phase II (research question 3). Following this design, the results from both phases are considered simultaneously to interpret the findings.

### 3.2. Procedures

Participants were 18 years or older, spoke fluent English, and were referred to a BIP as a requirement of a domestic violence charge. Men completed the surveys at their referral appointment with their probation officer before beginning the BIP in order to have a more representative sample (due to drop-out between time of referral and program intake). They completed the survey in a private office at the probation office, receiving $25 and light food/drink for their time. The original survey conducted from November 2017 to December 2018 consisted of 414 questions assessing men’s exposure to adverse childhood experiences (ACEs), mental health, physical health, attitudes, social networks, IPV, violence among non-intimate partners, and incarceration history (See [47]). For the current study, in Phase I, these participants were recruited between January 2019 and May 2019 from a sample of men who completed a broader survey on trauma and IPV (see [48]). Purposive sampling of men who endorsed key experiences (e.g., ACEs) on the survey were eligible for recruitment. At the time of the interview, all men were enrolled in the program (except 1 who dropped out), with participation in the program ranging from 3 to 36 weeks. In Phase II, a secondary data analysis was conducted using the original survey. For the purposes of the current study, data analysis focused on ACEs, aspects of mental health, social networks, and IPV. All study protocols were approved by the Institutional Review Board.

### 3.3. Sample

#### Phase I: Interview Sample and Phase II: Survey Sample

A total of 67 men participated in the original survey. Eight of the possible 67 completed a semi-structured interview, and three of these men consented to a follow-up interview, resulting in 11 semi-structured interviews. The mean age of the men was 35, with the majority being Black (Phase I: *n* = 7; Phase II: 76.1%), high school graduates (Phase I: *n* = 5; Phase II: 49.3%), employed (Phase I: *n* = 6; Phase II: 86.6%), and reporting less than $20,000 in annual income (Phase I: *n* = 8; Phase II: 71.6%). See Table 1 and Table 2 for complete demographic information.

### 3.4. Measures

#### 3.4.1. Phase I: Semi-Structured Interview

A semi-structured interview guide was created to illuminate participants’ narratives on how previous or current traumatic experiences and social support affected their relationships with other people, including intimate partners. For example, to cull men’s understanding of the impact of traumatic experiences on their relationships with others, we asked questions such as, “Do you think that some of the bad things that happened growing up and in adulthood impacted the way you treat or interact with others, like your intimate partner? How so?”

Participants were also asked about how their social networks affected their relationships with others, with questions such as, “Thinking about your family and friends, do you think that you interact with and receive enough support from them? If so, could you talk a little bit about how such strong ties with your family and/or friends affect your life in general?”, and, “How do you think the [support or lack of support] from your family and/or friends affects your relationship with your intimate partner?”

#### 3.4.2. Phase II: Survey

The metrics included below reflect a subset of variables measured in the original survey and used for the follow-up analysis during Phase II after the interviews.

**Demographics.** Race/ethnicity was measured using the following categories with the option to select more than one category: “White”, “African American or Black”, “Hispanic or Latino”, “Asian”, “Native American or Alaska Native”, and “other”. Income in the past year was measured using one item, with response options ranging from 1 = “less than $10,000” to 11 = “above $100,001”. Respondents indicated their level of education completed using the following options: “less than high school”, “some high school”, “high school diploma or GED”, “some college or college”, and “graduate school”.

**Adverse childhood experiences.** ACEs were measured by using the “traditional” 10-item ACE checklist (e.g., abuse, neglect, witnessing IPV, and household dysfunction; [52]) and an “extended” ACE checklist (11 items) that identify adversities that are systemic or in settings outside households (e.g., witnessing community violence, experiencing identity-based discrimination; [54,55]). The response option for each item was dichotomous, 0 = “*no*” and 1 = “*yes*”, making a total score ranging from 0 to 10 representing the number of “traditional” ACEs and a total score ranging from 0 to 21 representing the number of “traditional” and “extended” ACEs experienced by the respondent.

**Complex trauma symptomology.** Three subscales of the Structured Interview for Disorders of Extreme Stress-Self Report (SIDES-SR) measured complex trauma symptoms in the past month. Alterations in regulation of affect and impulses (17 items; total score ranging from 0 to 17) measures affect regulation, modulation of anger, and other destructive behaviors, which served as a proxy for the theme “Stifling Vulnerability and Emotions.” Serving as a proxy for the theme “Guilt and Shame,” Alterations in self-perception (6 items; total score ranging from 0 to 6) measures feelings of guilt, shame, and that “no one can understand.” Alterations in relations with others (5 items, total score ranging from 0 to 5) measures one’s “inability to trust,” victimizing, and victimizing others, which served as a proxy for the theme “Mistrust of Others.” Response options for each item were 0 = No, 1 = Yes. The total score for each subscale represents the count of trauma symptoms related to each subscale. This measure has shown good reliability and construct validity [56,57].

**Mental health.** The Mental Component Summary (MCS) score derived from the 13-item Health Status Survey Short Form Version 2 (SF-12v2) was used to measure men’s mental health. SF-12v2 uses Likert-type scales to measure the generic health status and has 8 subscales, each of which examines a domain of physical and mental health of individuals. The overall summary score of components related to mental health (i.e., MCS) was normed based on the population mean to allow for direct comparison of the MCS scores with the population mean (for more detailed explanations on the calculation of MCS, refer to [50]). The population mean is 50; therefore, any score above 50 is above the population mean [50]. Higher score of MCS indicates higher mental health quality of life. MCS has been reported to show good reliability and construct validity [58].

**Social network.** The 6-item Lubben Social Network Scale was used to examine the social network of individuals. Each item has 6 response options ranging from 0 to 5, each indicating the number of relatives and friends that the respondent can trust, call for help, or interact at least once during the month prior to the survey [51]. Each item score is summed to create a total scale score that could range from 0 to 30, with higher scores indicating greater levels of social support. A score less than 12 indicates that the respondent is at risk of experiencing social isolation [51]. The scale has been reported to show good reliability and construct validity [51].

**Intimate partner violence perpetration.** Thirty-three items from the Revised Conflict Tactics Scale (CTS2) measured men’s IPV perpetration, including psychological aggression, physical assault, injury, and sexual coercion [49]. Men reported frequencies of each behavior in the past year using a seven-point Likert scale (0 = “this has never happened”, to 6 = “more than 20 times”, and 7 = “not in the past year, but it did happen before”). Instructions from Straus and colleagues were followed to create frequency scores in the past year for each subscale and a “total” frequency score of all subscales [49]. Following author instructions [49], severity categories (i.e., none, mild, and severe) were also created for each eligible subscale. This measure has demonstrated good validity [59] and reliability [49] in previous studies.

### 3.5. Analysis Plan

#### 3.5.1. Phase I: Qualitative Analysis

Research questions 1 (RQ1) and 2 (RQ2) were answered using narrative analysis. Narrative analysis gathers individual stories told by a small number of participants and reorganizes them in a chronological order to create an overarching framework based on common significant life experiences identified across individual stories (i.e., “turning points”; [60]). Turning points were coded within each participant’s story that show how childhood trauma and men’s social support affects their relationships with other people, including intimate partners. Thereafter, turning points from each person’s story were reorganized into a chronological order to illuminate common pathways explaining how childhood trauma and social support have affected men’s relationships with others. Each participant was assigned a pseudonym to ensure anonymity.

**Rigor.** To enhance the rigor of the qualitative analysis, the second author debriefed with the research team after each interview to discuss noteworthy findings and strategize his approach for future interviews. The second and third author who received doctoral-level training on qualitative methods separately coded the transcripts and had subsequent meetings with each other and the first author to check the accuracy of the coding process and come to consensus on the final codes to be derived from the transcripts. Lastly, to enhance the validity of the findings identified from the reorganized stories, three of the eight participants provided feedback on the framework, assessing it for accuracy and ensuring that the framework aligned with their life experiences.

#### 3.5.2. Phase II: Quantitative Analysis

Univariate analyses were conducted for each variable. Bivariate associations between each variable were examined with Pearson’s correlation analysis, and a series of Ordinary Least Square (OLS) regression models were used to answer research question 3 (RQ3).

## 4. Results

### 4.1. Men’s Collective Narrative

Setting the foundation for adult relationships, some men experienced severe forms of abandonment or betrayal in childhood, particularly sexual abuse, and perceived pressure to not disclose these experiences due to stigma. Other men also perceived a social pressure in childhood and adolescence to “not look weak” by sharing their emotions. These developmental life experiences shaped men’s world view, pressuring them to “look strong” by not showing emotions, making them mistrust others and develop a sense of guilt and shame about themselves. These underlying mechanisms were then carried forward into adult relationships where men adopted social isolation as a coping strategy to manage adversity and trauma in adulthood, which led to further deterioration of men’s relational and mental health, affecting their relationships.

### 4.2. Shaping Men’s World View: Trauma and Lack of Relational Support (RQ1)

Five men shared experiencing significant adversity or trauma such as abuse and loss of loved ones. For some cases, these traumatic experiences were paired with abandonment from close family members or intimate partners at times of struggle, which contributed to men’s tendency to view people around them as generally unreliable. For example, Chris is a 49 years old, Black man, reporting an annual income less than 20k/year, with a college degree. He described that being abused by those around him has been a “constant theme” in his life. He explained feeling inadequate relational support in that his family and intimate partner took advantage of and abandoned him when he was suffering from the loss of his father. The experience of being taken advantage of and abandoned in addition to being victimized led him to perceive the world as a “scary place” with “awful people” in it.

“I’m still kind of processing and mourning and dealing with a dysfunctional partner and I have been fighting depression, doing the best I can, but, I’ve also been victim to more mental abuse and torture and so... it’s like a bad movie. … I’m thinking this is what a significant other does, is support, they help, they’re there with you. They’re fighting with you to get you better, to get you to where you need to be at. I’m looking back now and I’ve never had that from a significant other. I’ve never had it. In thirty years of being out in the world and dating and relationships and seeking that mate that could give me that, but... it’s been a learning experience, though. Just this past couple months, or since my dad’s been [dead]—I’ve grown up a lot, you know? The world’s a big, scary place and there’s some awful people in it. You know, there’s good people, too, but you’ve got to be careful. You’ve got to be careful. You can’t be vulnerable, because people will take advantage of you if they can.” (Chris)

The feeling of abandonment was not solely experienced at the interpersonal level. Michael, an 18-year-old Black man, reporting no annual income and completing some high school, told a narrative which illuminated the larger cultural or societal norms affecting him by way of heightened fear of stigmatization and marginalization, contributing to a diminished sense of relational support. Specifically, Michael described feeling social pressure as an African American man to hide his history of sexual abuse due to the stigma in his community that boys who are sexually victimized by another man are labeled homosexuals. The fear of being stigmatized primed him to feel that nobody, not even his own family, would ever be there to support him.

“I told you I was sexually molested by one of my family close friends or whatever. And I feel like if I ever told any other soul in my family I knew for a fact I would be looked down bad. But, it’s not that I didn’t want to, you know, because my family, you know, is a Black, is, you know, African American family, you feel me? And you know, the African American community, they really don’t [sic] look down on gay people, whatever. And I’m not [gay], you feel me? I just got molested. And I was scared. I didn’t know what to do, you feel me? I was scared to tell anybody because I know for a fact how my family is...I would never hear the end of it for the rest of my life. You gay this, this that and the third. But really, I was just thinking like, nobody was there for me so from that point on I always thought that nobody would be there for me.” (Michael)

The combined effects of traumatic experiences and receiving insufficient amount of support, care and understanding from others shaped men’s views of how others would treat them throughout their lives, contributing to their decreased sense of relational support in general.

### 4.3. Unresolved Trauma and Maladaptive Coping Strategies in Adulthood (RQ2)

In the larger sample of the current study, nearly 30% of men were considered socially isolated (see Table 2). In the semi-structured interviews, men were asked to reflect on their social networks. Seven of the eight participants expressed feeling a certain level of social isolation in adulthood. For example, Anthony (a 29-year-old Black man, reporting earning less than 20k/year, with a GED/HS degree) describes with surprise how he does not have friends outside of his family.

“It seems crazy to be thirty and I’m like, if I threw a birthday party this summer, like, who would be there? It would just be family, you know?” (Anthony)

Chris described being saddened by a lack of friends to rely on for support as a middle-aged man.

“It’s sad. I don’t have a relationship, I don’t have any friends that I trust, you know? I mean, but I knew that. But you asking me having to say it out loud to somebody, even somebody who I don’t know, it’s a hard pill to swallow. That a fifty-year-old man can’t say he has one friend in this whole world that he can go to now and say, hey, I trust you. I need to talk to you. I need to be with you.” (Chris)

Stressors in adulthood such as emotional or financial challenges also contributed to isolation of several men. For example, Jamar (34-year-old Black man, reporting earning less than 20k/year, with a college degree) described that he did not want to talk about his feelings with others when he was feeling depressed and would just “hide away” from his family and friends. Cameron (a 28-year-old Black man, reporting earning less than 20k/year, with a GED/HS degree) described isolating himself when having financial challenges to figure out “how to get money”.

Men who isolated themselves described a dissatisfaction or feeling of sadness about their isolated status. The question is, then, why these men chose isolation as a coping strategy when they find it maladaptive. Narratives told by men further illuminated several mechanisms leading to participant’s social isolation and how isolation affected their relationships with other people, particularly with intimate partners.

**Mistrust of others.** Four men shared a sense of mistrust of others and mentioned that as a result of mistrust, they refrained from closely interacting with others and isolated themselves when renewed experiences of betrayal and abandonment emerged in adulthood. For example, Tyler (a 31-year-old Black man, reporting earning less than 20k/year, with a GED/HS degree) described experiencing an “ultimate betrayal” from his previous intimate partner who cheated on him and shared his “dark secrets” with the other man. Tyler says that he “should have never told her anything”, and he also reported that he expects “nothing at all” from his family members because they had not shown him the level of care and favor that he showed them. When asked about how he reacts to the experiences of betrayal from others like these, he reported that he would isolate himself.

“[When feeling betrayed] I want to go, like, isolate myself from people. Like, I kind of like, been doing a good job at it, too.” (Tyler)

Anthony said that he does not hang out with friends and isolates himself because his friends “tarnished” his relationship with his intimate partner and also tried to “keep him down” in negative environments and moods. He goes on to explain that people are only friends with him for personal gain, such as when he suggests that his friends were trying to “get” his then girlfriend.

“That was something I had to learn, too, because people will be haters. They will see you being happy and it wasn’t necessarily [obvious], but it would be like, you know, we all chilling, I got my lady with me, and somebody would make a inappropriate joke. It’s, like, c’mon, like, you see my lady right here, why would you even bring the subject up or why would you even start talking about that? And I didn’t realize [it] before, but I realize it now. Like, they were on the low hate, you know what I’m saying? On the low trying to get her. … I think having friends in that early relationship, that’s, I think that did mess up my relationship with my daughter’s mother … I don’t think [having friends around] matters that much because people only going to be around you for what they can get from you, what they can get out of you, what they can gain from the situation … So, and I know that. And if my friends, they want to hold me down, they want to, you know what I’m saying, keep me down, I just can’t run with it, you know?” (Anthony)

James is a 40-year-old Native American/Alaskan Native man, who reported earning less than 10k/year, with a GED/HS diploma. He also reported he mistrusted people after having issues with friends, which contributed to his tendency to isolate himself.

“I don’t trust too many people at all … As far as friends goes, I never really had, I’d never really allow myself to have too many friends, because I didn’t know what they were being my friend for, you know? … So, I kind of like, isolate myself. You know what I mean? Not on purpose, I just don’t feel like being bothered, you know?” (James)

Taken together, trauma and adversity paired with a lack of social support contributed to men’s mistrust of others in general, shaping their tendency to choose isolation as a strategy to cope with stressors in adulthood.

**Stifling Vulnerability and Emotions.** Notably, men did not explicitly discuss the association between childhood experiences and their tendency to isolate themselves as coping mechanisms for stress in adulthood; however, Jamar describes how his father raised him to “hold in” his emotions which led him to restrict his emotions with friends. He describes how his limited ability to rely on others in times of need can be emotionally overwhelming.

“I’m the strong person. I need to hold it in. I need to be the example. That’s how I was raised. So, I didn’t know how to really talk to nobody when I had issues or problems or emotions or say when I’m sad or hurt. No, I didn’t know what that meant. I was that rock, you know? I was supposed to be that strong one and can accomplish anything, you could deal with any situation.” (Jamar)

“Ever since I was younger, and I just kept, I keep things in. I don’t express myself as much as I should. I’ve looked at it as being weak or didn’t want to expose myself or felt, put a persona on, but, it was hard for me for a while to express and touch or, and talk about things that may happen to me or bothered me … I had a lot of pressure. I was always the leader, I was always the person to look up to and depend on and that’s been mostly, basically the story of my life. Even friends. It just became overwhelming, you know, I think now I’m looking back at it. Even friends found, I mean, it was amazing. It’s amazing. It was overwhelming.” (Jamar)

Men also expressed a fear of being taken advantage of, encouraging them to stifle their vulnerability. Three men described that they were taken advantage of in close relationships, such as intimate partners, once they expressed their vulnerability with them. For example, Tyler said that his intimate partner shamed him for his “dark histories” he shared with her and later cheated on him, which he described as a “betrayal”. Chris said that, although he does not try to suppress his emotions, he recognizes that letting “too much be shown” lets other people take advantage of him.

“I try to pride myself on [being able to open up emotionally] although now I’ve realized it’s gotten me in trouble, because I’ve shared too much and I let too much be shown. I think sometimes you can be too raw with someone and they can take that and use that to their advantage.” (Chris)

These narratives suggest that the socially learned or prescribed masculine norms of emotional suppression and experiences of being taken advantage of when showing their vulnerability to others may have led these men to further refrain from expressing any vulnerable emotions. As a result, these men tended to isolate themselves rather than talking with others about their struggles when feeling stressed.

**Guilt and Shame.** Two men also described that guilt and shame served as a reinforcing mechanism that prevented them from sharing their emotions during times of great stress in adulthood, prompting social isolation. Tyler said that he did not express his feelings of stress resulting from adversities in adulthood such as incest because he felt guilty and ashamed.

“I mean, you’ll be the first person outside of my wife and pastor, I told you, I told you. But, my family...it’s incest. Like, that shit rampant in my family. That’s why I don’t like talking about it. You get what I’m saying? That shit ain’t normal. … It’s just that, like, it feels shameful. Like, I feel kind of guilty. Like, it’s your fault.” (Tyler)

James described that, although he is now able to open up emotionally after receiving therapies, he used to feel stigmatized based on the adversities he faced in adulthood such as homelessness. This stigma made him fear how others would judge him and subsequently held his feelings in.

“I slept on porches in the wintertime, I done walked around all day because people, you know, they looking at me all funny. They’re not saying they don’t want me around, but I could feel the energy like, oh, man, he around. He’s about to be asking for stuff... … And I just learned [in therapy] how to open up and just express myself. So, no, I’m not influenced [by what others think of me], like, I feel what I feel. I’m not going to hold it in because of what somebody else would think. They’re going to judge me like this, they’re going to judge me like that, they’re going to think about me like this, they’re going to think about me like that. I don’t [hold in my emotions] anymore. I have, in the past, you know, but not no more.” (James)

Taken together, men’s past adversities and social pressure at the family, peer and societal level prompted them to restrict their emotions and isolate themselves.

**Mental health.** The lack of social support in adulthood may have a detrimental impact on their overall mental health, deteriorating the emotional instability of those who were already suffering from significant stress. Chris mentioned that he has suffered from frustration and depression since being abandoned from his family members and intimate partners after his father passed away, a person whom he considered one of his few social supporters. Jamar also described how his social isolation contributed to his increased dependence on alcohol and drugs. In a relatively extreme case, Tyler even experienced auditory hallucinations and even started to harm himself as he continued isolating himself:

“But, no, [isolating myself is] not healthy, though, because that isolation could lead to other stuff, you know, me getting trapped in my thoughts, and I start hearing them voices again. Hearing them voices I start hurting myself and doing little weird stuff and I don’t want to do all that.” (Tyler)

### 4.4. Phase II: Quantitative Follow-Up (RQ3)

Based on men’s narratives, bivariate associations were examined to assess if the relationships described in men’s narratives were evident in the larger sample and if they were significantly correlated with IPV perpetration in the past year. Following this, we then ran simplified OLS regression models using “total IPV frequency” as the outcome variable to assess if the associations held (see Table 3).

### 4.5. ACEs/Trauma, Social Support, and IPV

Men in the study reported an average of 3.5 (SD = 2.66) ACEs on the original ACEs Checklist and an average of 8.58 (SD = 4.96) ACEs on the expanded ACEs checklist (See Table 2). The association between ACEs that were reported in the collective narrative (i.e., sexual abuse, not feeling loved, not feeling protected in childhood, physical abuse), IPV in adulthood, and their self-reported level of social support were examined. Results showed that physical abuse (*r* = −0.277, *p* < 0.05) and “not feeling loved” (*r* = −0.343, *p* < 0.01) were negatively associated with social support in adulthood. Considering the associations between ACEs and IPV perpetration, sexual abuse was positively associated with psychological aggression (*r* = 0.285, *p* < 0.05) and IPV injury (*r* = 0.373, *p* < 0.01). “Not feeling loved” was positively associated with psychological aggression (*r* = 0.426, *p* < 0.01) and physical IPV (*r* = 0.270, *p* < 0.05). “Not feeling protected” was positively associated with physical IPV (*r* = 0.344, *p* < 0.01) and IPV injury (*r* = 0.380, *p* < 0.01). Results from the OLS model indicated that the increased report of traditional 10 ACEs and the decreased report of social support significantly predicted increased overall IPV frequency (*p* = 0.007). At the predictor level, men reporting a greater number of traditional ACEs were more likely to report greater levels of IPV perpetration, controlling for social support. When modeling the extended ACEs checklist with social support, this model was not significant.

### 4.6. Maladaptive Coping Strategies in Adulthood and IPV

**Mistrust of others.** Half of the men in Phase I explicitly acknowledged that their mistrust of others impacted their relationships in adulthood. In Phase II, “Alterations in relationships with others” (a proxy measure for mistrust of others) was negatively associated with men’s social support (*r* = −0.352, *p* < 0.01), and it was positively associated with men’s perpetration of psychological aggression (*r* = 0.539, *p* < 0.001), physical assault (*r* = 0.280, *p* < 0.05), and IPV injury (*r* = 0.339, *p* < 0.01). Results from the OLS model indicated that together, men’s increased symptoms of “Alterations in relationships with others” and decreased social support significantly predicted higher frequency of IPV perpetration (*p* = 0.006). At the predictor level, men experiencing greater level of “Alterations in relationships with others” were more likely to report higher frequencies of IPV perpetration, controlling for social support.

**Stifling Vulnerability and Emotions.** Men’s collective narrative described how they stifled their emotions to protect their vulnerability, but this led to isolation as a coping mechanism during stressful times. The survey data from the larger sample showed that the severity of the men’s trauma symptoms is negatively associated with the level of their social support (*r* = −0.419, *p* < 0.01), indicating that men with greater trauma may be more likely to be socially isolated. Bivariate associations in the larger sample show that men’s “Alterations in regulation of affect and impulses” (e.g., modulation of anger) was positively associated with psychological aggression (r = 0.403, *p* < 0.01). Results from the OLS model indicated that increased symptoms of “Alterations in regulation of affect and impulses” and decreased social support together significantly predicted higher rates of IPV perpetration (*p* = 0.013). At the predictor level, men experiencing greater “Alterations in regulation of affect and impulses” were more likely to report a higher frequency of IPV perpetration, controlling for social support.

**Guilt & Shame**. Men described how their guilt and shame served as a mechanism to hide their emotions and to isolate themselves. According to the bivariate analysis, men who reported greater social support were also less likely to report complex *Alterations in self-perception*, particularly related to guilt and shame, in the past month (*r* = −0.453, *p* < 0.001). In turn, men’s “Alterations in self-perception” were significantly associated with IPV psychological aggression (*r* = 0.668, *p* < 0.001) and injury (*r* = 0.431, *p* < 0.01) perpetration. Results from the OLS model indicated that increased symptoms of “Alterations in self-perception” and social support significantly predicted higher rates of IPV perpetration among men (*p* < 0.001). At the predictor level, men experiencing greater levels of “Alterations in self-perception” were more likely to report higher rates of IPV perpetration, when controlling for social support.

**Mental Health.** Some men described how their mental health impacted their social and intimate relationships in the narrative analysis; the relations between social networks, mental health, and IPV were validated in the larger sample. That is, men who reported larger social networks were more likely to report positive mental health well-being (*r =* 0.284, *p* < 0.05). In turn, men’s self-rated mental health well-being was significantly associated with decreased psychological aggression (*r =* −0.433, *p* < 0.01), physical assault (*r* = −0.333, *p* < 0.05), and injury (*r =* −0.384, *p* < 0.01) perpetration. In the OLS model, men’s report of lower levels of mental health and social support significantly predicted higher rates of IPV perpetration (*p* = 0.022). At the predictor level, men reporting lower levels of mental health well-being were more likely to report greater levels of IPV perpetration, controlling for social support.

## 5. Discussion

This study employed a novel, exploratory design to identify potential factors stemming from childhood that were reinforced throughout the life course, which contributed to the relational development of BIMOC with criminal justice involvement. To our knowledge, this study is the first to explore the effects of social support on socioeconomically disadvantaged men’s IPV perpetration and adds to the growing body of literature examining the effects of ACEs and trauma on men’s IPV perpetration.

Men in this study reported higher rates of childhood adversity and trauma compared to men in the general population [52], which aligns with previous research documenting disparities across race/ethnicity and income status [61]. Although previous research has linked ACEs with men’s IPV perpetration [32], men’s narratives in the current study illuminated certain forms of adversity and trauma in childhood that were salient to BIMOC’s lived experiences (namely physical and sexual abuse, not feeling loved, and not feeling protected). Adding to the body of literature examining the effects of childhood abuse and neglect on interpersonal violence in adulthood [62], this study highlights the powerful effects of less commonly studied forms of adversity, namely children not feeling loved or protected.

The link between ACEs and IPV perpetration is not deterministic and must be considered in the context of other risk and protective factors. To that end, the majority of men in the qualitative sample described how the combined effect of traumatic experiences and a lack of relational support from family shaped their worldview to expect that no one would be there for them (for example, when Michael disclosed keeping his childhood sexual abuse secret from his family for fear of ridicule). Similar research has found that “betrayal trauma” (or early experiences of violation perpetrated by a close, trusted person) can have significant negative effects on an individual’s trust in others, thus impacting their future social relationships [63]. Previous studies suggest that individuals with high levels of betrayal trauma in childhood have lower levels of self-reported general and relational trust in young adulthood, which may increase their chance of revictimization in interpersonal relationships [64]. The current study suggests that without sufficient relational support for boys, the impact of early trauma may also be a salient experience for the risk of future IPV perpetration among BIMOC.

Aligning with Trauma Theory, the worldview imparted from early experiences of trauma and adversity shaped BIMOC’s relationships with others. This internal schema developed in childhood led men to use social isolation as a coping mechanism in adulthood that, in turn, adversely affected their interpersonal relationships (such as when Tyler described feeling betrayed and isolating himself as a result). We identified three underlying mechanisms that potentially reinforced men’s early worldviews and perpetuated the use of isolation as a coping strategy: namely, the mistrust of others, stifling vulnerability and emotions, and guilt and shame. This narrative was supported in the quantitative follow-up (i.e., Phase II) with significant bivariate associations and OLS regression models found between each of these potential mechanisms, low social support, and increased IPV. Similarly, researchers examined maladaptive interpersonal styles, negative experiences in childhood, and core self-schemas in a study of 300 nonclinical adults (42% men; [65]). Researchers reported that “Disconnection and Rejection” was a significant antecedent to maladaptive interpersonal styles [64], which is similar to the men in the current study who reported trauma and a lack of relational support (either through abandonment or betrayal or to self-imposed for protection) as salient experiences in childhood.

Additionally, the current study’s findings indicated that the schemas adopted by men (i.e., mistrust of others, stifling vulnerability and emotions, guilt and shame) were associated with less social support (a maladaptive coping mechanism identified by men) and increased IPV. These results align with and add to the limited body of research unpacking the underlying mechanisms of IPV perpetration. For example, LaMotte and colleagues found that mistrust of others mediated the relationship between experience of trauma and intimate partner aggression among 83 heterosexual couples [66]. Tezel and colleagues reported that the early maladaptive schemas mediated the relationship between (1) childhood sexual abuse and “Emotional Avoidance” and “Manipulative and Abusive” interpersonal styles in adulthood; the relationship between (2) childhood emotional abuse and “Emotionally Avoidant” and “Avoidant” interpersonal styles in adulthood; and the relationship between (3) childhood physical abuse and “Avoidant” and “Abusive Interpersonal” styles in adulthood [65]. The one incongruent finding in the OLS regression models was that increased social support and increased “Alterations in self-perception” significantly predicted higher rates of IPV perpetration. Given that guilt and shame are key symptoms of “Alterations in self-perception”, it is possible that increased social support may exacerbate these symptoms and trigger the use of IPV. Finally, men in the current study described how their social isolation exacerbated their overall mental health, such as Chris’ depression, Jamar’s substance abuse, and Tyler’s auditory hallucinations. Their involvement in IPV may have also been further triggered by their isolation, considering that deteriorated mental health significantly increases the risk of IPV perpetration [29].

## 6. Limitations

Though the collective narrative shared by men was validated in the larger sample, the results stemming from these men’s experiences may not be transferable (in the qualitative paradigm) or generalizable (in the quantitative paradigm) to other populations. Caution should be used when considering nonmandated clients or clients who are not of low-income or identify as BIMOC. Phase II of the study was limited in sample size and statistical power to test the associations with more advanced models that included key covariates (such as age) or a sophisticated analytic procedure such as structural equation modeling. The study aims were met given that the study was exploratory; however, further research with larger samples including clinical and nonclinical populations, and more sophisticated analyses are needed to validate the study findings. Finally, we chose to model “total IPV frequency” rather than modeling each subtype for the OLS regression models to simplify the results for interpretation and to reduce the risk of Type I error; however, from a clinical perspective, total frequency is less meaningful because there is no true “zero” because the sample is made up of men who have perpetrated at least some IPV. Emphasis of these findings should be placed on the confirmation of the relationship.

### 6.1. Practice Implications

Batterer interventions are an existing structure to develop social support among men. In their current form, most BIPs focus on creating honest, self-disclosing intimate relationships among men. Building from the strength of current programming, BIPs could be augmented to enhance men’s relationships with each other in a number of ways. First, practitioners should screen for social support, social isolation and mental health problems to identify high-risk clients and target resources and referrals as appropriate. As practitioners work with men at risk for IPV perpetration, they should consider the facets of the Relational Cultural Model of Development and the corresponding Relational Cultural Therapy (RCT; [67]) to build relational health among men. RCT posits that people grow through and toward relationships throughout the lifespan; that relationships built on mutual empathy foster a greater sense of connection; and that chronic disconnection can lead to social isolation [68]. Centralizing relationships is a key mechanism to men’s healing via the practitioner–client relationship, peer relationships among men in the group, and support for men’s relationships with family, friends, and intimate partners. In these efforts, practitioners should consider how the key factors underlying men’s social isolation, namely mistrust of others, guilt/shame, and stifling vulnerability manifest in their relationship with the client, client’s relationship with other men in the group, and their intimate partner. Within this context, practitioners need to consider how BIMOC’s sociocultural experiences interact with these factors and shape relationships—for example, considering how structural racism may affect BIMOC’s mistrust of others and the necessity they feel to stifle vulnerability. Notably, RCT may not be appropriate for all men, such as men with severe mental health issues. Additionally, peer-mentoring models could be incorporated into programming with men enrolled in the BIP. Finally, peer support groups could be offered post-program completion with minimal supervision by program staff.

### 6.2. Future Research

Future qualitative studies should build upon this exploratory research, and quantitative studies should confirm narratives of men with similar early life experiences who did not perpetrate IPV to identify protective factors that may mitigate this pathway. Qualitative studies should explore existing sources and potential avenues to enhance social support (and other forms of relational health) among adolescent boys and men, especially among those exposed to adversity and trauma. Given the critical context of structural racism, these studies should also explore how sociocultural experiences interact with relational health among adolescent boys identifying as Black, Indigenous, and People of Color, with varying income statuses. Quantitative studies with larger samples should test the protective effects of social support (and other forms of relational health) on men’s risk of IPV perpetration. Furthermore, researchers should design studies with attention to how development may affect the relationship between men’s relational health, maladaptive coping strategies, and IPV. Finally, the moderating effect of social isolation on the relation between men’s mental and emotional health and IPV perpetration should be examined in quantitative studies with larger samples.

## Figures and Tables

**Table 1:** Qualitative Study Sample Demographics, ACEs, IPV Perpetration Severity, and Mental and Relational Health Variables (*N* = 8).

Name	Demographics				ACE	IPV Perpetration Severity (Past Year) ^a^				Mental and Relational Health				
	Age	Race	Annual Income	Education		Psych	Phys	Sexual	Injury	AR ^b^	SP ^b^	RO ^b^	MCS ^c^	Social Isolation ^d^
Jamar	34	Black	$10,001–20,000	College	4	Severe	Mild	Mild	None	4	0	1	51.12	NI
Cameron	28	Black	$10,001–20,000	HS/GED	1	Severe	Mild	Mild	Mild	2	0	0	64.48	NI
James	40	NA/AN	<$10,000	HS/GED	6	Severe	Mild	Mild	None	1	2	3	52.97	NI
Tyler	31	Black	$10,001–20,000	HS/GED	7	Severe	Severe	Severe	None	4	4	3	36.13	At risk
Anthony	29	Black	$10,001–20,000	HS/GED	6	Mild	None	None	None	0	0	0	41.76	NI
Chris	49	Black	$10,001–20,000	College	4	Severe	Severe	Severe	None	0	0	0	44.47	NI
Michael	18	Black	None	Some HS	4	Severe	Mild	Mild	Mild	5	4	2	37.22	N/A
Isiah	50	Black	$10,001–20,000	HS/GED	0	Severe	None	None	None	0	0	1	58.32	At risk

AR = alterations in regulation of affect and impulses. SP = alterations in self-perception. RO = alterations in relations with others. MCS = Mental Component Summary. NI = Not isolated. NA/AN = Native American or Alaska Native. HS/GED = High school diploma or GED. At risk = At risk of social isolation. Some HS = some high school. N/A = Not applicable (data missing). ^a^ IPV perpetration severity categories were created based on guidelines suggested by Straus and colleagues [49]. ^b^ Count of trauma symptoms that correspond to the alteration category. ^c^ Higher MCS scores indicate higher mental health quality of life, and scores above 50 are above the population mean [50]. ^d^ Lubben Social Network Scale score less than 12 indicates that the respondent is at risk of experiencing social isolation [51].

**Table 2:** Quantitative Study Sample Demographics, ACEs, IPV Perpetration Past Year Frequency, and Mental and Relational Health Variables (*N* = 67).

Characteristic/Variable.	*N* (%)	*M (SD)*	Clinical Threshold (%)	Min	Max
Age (*N* = 67)		35.42 (11.61)		18	67
Race (*N* = 67)					
White	4 (6.0)				
African-American or Black	51 (76.1)				
Hispanic or Latino	7 (10.4)				
Native-American or Alaska Native	1 (1.5)				
Other	4 (6.0)				
Annual income (*N* = 66)					
Less than $10,000	21 (31.3)				
$10,001–$20,000	27 (40.3)				
$20,001–$30,000	10 (14.9)				
$30,001–$40,000	3 (4.5)				
$40,001 or higher	5 (7.0)				
Education level completed (*N* = 66)					
Less than high school	3 (4.5)				
Some high school	18 (26.9)				
High school diploma or GED	33 (49.2)				
Some college or college	12 (17.9)				
IPV Perpetration					
Psychological aggression (*N =* 58)		31.91 (30.47)		0	116
Physical assault (*N =* 57)		6.25 (12.96)		0	84
Injury (*N =* 61)		3.80 (7.63)		0	31
10 Traditional ACEs (*N* = 66)		3.50 (2.66)	51.5 ^a^	0	9
21 Extended ACEs (*N* = 66)		8.58 (4.96)		0	20
Trauma Symptomology					
Affect and impulse regulation (*N* = 56)		2.70 (2.52)	60.7 ^b^	0	9
Self-perception (*N* = 62)		1.34 (1.64)	35.5 ^b^	0	6
Attention or consciousness (*N* = 63)		1.27 (1.46)	33.3 ^b^	0	5
Somatization (*N* = 67)		1.60 (1.91)	38.8 ^b^	0	6
Systems of meaning (*N* = 63)		1.00 (1.28)	25.4 ^b^	0	4
Relations with others (*N* = 61)		1.66 (1.50)	45.9 ^b^	0	5
Mental Component Summary (*N* = 60)		−0.15 (1.12)		−3.86	1.66
Social Network (*N* = 60)		14.92 (6.98)	29.9 ^c^	0	30

^a^ Four or more ACEs were used as a threshold above which there is a higher risk of negative developmental outcomes [52]. ^b^ SIDES-SR subscale score greater than 2 were considered to be indicative of clinically significant cases for that domain [53]. ^c^ Lubben Social Network Scale score less than 12 indicates that the respondent is at risk of experiencing social isolation [51].

**Table 3:** OLS Regression Models of ACEs, Maladaptive Coping Strategies, and Total IPV Perpetration Frequency.

Model	*R* ^2^ *_adj_*	*B*	*SE_B_*	*β*	*t*	*sr* ^2^
**Model 1-A: ACES (Traditional-10), Social Support, and IPV (*N* = 59)**						
	0.14 **					
(Intercept)		63.55	31.46		2.02	
ACEs (10 items)		12.12 **	3.93	0.40	3.08	0.38
Social support		−0.20	1.50	−0.02	−0.13	−0.02
**Model 1-B: ACES (Expanded–21), Social Support, and IPV (*N* = 42)**						
	0.10					
(Intercept)		68.99	38.44		1.79	
ACEs (21 items)		5.97 *	2.60	0.35	2.30	0.34
Social support		−0.90	1.76	−0.08	−0.51	−0.08
**Model 2: Alterations in relationships with others, Social Support, and IPV (*N* = 57)**						
	0.14 **					
(Intercept)		69.59	30.35		2.29	
Alterations in relationships with others		21.88 **	7.04	0.41	3.11	0.38
Social support		−0.19	1.52	−0.02	−0.12	−0.02
**Model 3: Alterations in regulation of affect and impulses, Social Support, and IPV (*N* = 52)**						
	0.13 *					
(Intercept)		78.20	30.49		2.56	
Alterations in regulation of affect and impulses		12.44 **	4.33	0.39	2.88	0.38
Social support		−0.58	1.56	−0.05	−0.37	−0.05
**Model 4: Alterations in self-perception, Social Support, and IPV (*N* = 57)**						
	0.28 ***					
(Intercept)		52.12	27.25		1.91	
Alterations in self-perception		28.28 ***	6.10	0.57	4.64	0.53
Social support		0.88	1.43	0.08	0.61	0.07
**Model 5: Self-rated mental health well-being, Social Support, and IPV (*N* = 57)**						
	0.10 *					
(Intercept)		232.501	46.48		5.00	
Self-rated mental health well-being		−2.48 *	0.95	−0.34	−2.61	−0.33
Social support		−0.60	1.53	−0.05	−0.39	−0.05

*Note.SE*_B_ = Standard error of coefficient. *sr*^2^ = Squared semi-partial correlation. *R*^2^*_adj_* = Adjusted R^2^. * *p* < 0 05. ** *p* < 0.01. *** *p* < 0.001.

## Data Availability

The data in this study are available upon request from the corresponding author. The data are not publicly available due to privacy restrictions.
